# Effects of Xanthan Gum, Lambda-Carrageenan and Psyllium Husk on the Physical Characteristics and Glycaemic Potency of White Bread

**DOI:** 10.3390/foods11101513

**Published:** 2022-05-23

**Authors:** Zawanah Yassin, Yin Li Tan, Akila SRV, John Monro, Lara Matia-Merino, Kaiyang Lim, Allan Hardacre, Suman Mishra, Kelvin Kim Tha Goh

**Affiliations:** 1 Singapore Institute of Technology-Massey University Food Technology, Dover Campus, 10 Dover Drive, Singapore 138683, Singapore; zawanahyassin@gmail.com (Z.Y.); 1701030@sit.singaporetech.edu.sg (Y.L.T.); 2 School of Food & Advanced Technology, Massey University, Private Bag 11222, Palmerston North 4410, New Zealand; akila.srv@csiro.au (A.S.); l.matia-merino@massey.ac.nz (L.M.-M.); a.hardacre@massey.ac.nz (A.H.); 3 CSIRO, Agriculture and Food, 39 Kessels Road, Coopers Plains, QLD 4108, Australia; 4 The New Zealand Institute for Plant and Food Research Limited, Private Bag 11600, Palmerston North 4442, New Zealand; john.monro@plantandfood.co.nz (J.M.); suman.mishra@plantandfood.co.nz (S.M.); 5 ES-TA Technology Pte Ltd., 21 Jalan Mesin, Singapore 368819, Singapore; kaiyang.lim.john@gmail.com

**Keywords:** glycaemic potency, nonstarch polysaccharides, starch–NSP interactions, bread, psyllium husk, starch digestion

## Abstract

White bread contains a high proportion of easily digestible starch, which contributes to an undesirable rapid increase in blood glucose concentration. This study investigated the effects of nonstarch polysaccharides (NSP) -xanthan gum, lambda-carrageenan and psyllium husk on the physical functionality and glycaemic potency of white bread. The amount of water for each formulation was adjusted based on DoughLab set at a target torque value of ~500 FU for sufficient dough development. Adding NSP generally resulted in significantly increased loaf volumes and decreased hardness. The glycaemic potency (glycaemic glucose equivalents (GGE) g) of bread was found to be reduced with the addition of NSP at all levels (1, 3 and 5% *w*/*w* based on flour weight). Increasing the concentration of xanthan gum and lambda-carrageenan did not show any further decrease in the glycaemic potency. Notably, adding 5% *w*/*w* psyllium husk significantly reduced the glycaemic potency from ~49 GGE/100 g in the reference bread to 32 GGE/100 g. The reduction in the glycaemic potency was attributed to viscosity effects (for xanthan) and starch–NSP interactions (for psyllium husk). Overall, the 5% *w*/*w* psyllium husk bread sample was most promising in terms of both physical characteristics and its effect on in vitro glucose release.

## 1. Introduction

The prevalence of Type 2 diabetes mellitus has been increasing globally over the last decade. In 2021, the International Diabetes Foundation reported that more than 5.3 million adults (20–79 years) are living with Type 2 diabetes. This number is envisaged to rise to 783 million by 2045 (International Diabetes Federation, 2021). Furthermore, diabetes and glucose intolerance are associated with a marked increase in severity of response to COVID-19 infection and a poor prognosis [1]. Characterised by abnormally high levels of glucose in the blood, diabetes is a chronic condition that can be managed by controlling the amount of easily digested starch in the diet, to keep the blood glucose levels under control.

The potential of food to elicit a glycaemic response is termed its glycaemic potency [2]. Consumption of carbohydrate-rich foods is known to induce a glycaemic response, which is an increase in blood glucose concentration. The extent of glycaemic response to a food matrix is affected by the assembly of components present in the food matrix, such as rapidly digestible carbohydrate, fat, protein, dietary fibre, organic acids and phytochemicals [3]. Therefore, modifying the physico-chemical properties of a food matrix can potentially alter its rate of digestion, which also affects the rate of gastric emptying and nutrient absorption [3]. 

Bread is a starch-rich staple food usually formulated from a mixture of water, wheat flour and yeast. The ingredients are mixed and kneaded to develop a gluten structure (dough), followed by fermenting (proofing) and baking the viscoelastic dough. The foam-like bread crumb structure in bread is attributed to the incorporated air during mixing, CO_2_ formed during fermentation within the elastic gluten matrix and water vapour from steam during baking. The starch in wheat flour acts as a filler in the continuous gluten matrix [4,5]. About 85% of the starch found in bread is considered to be rapidly digestible starch (RDS) due to the gelatinisation of starch during the baking process (≥70 °C) at relatively high moisture content (≥35%) of the dough [6]. Fully gelatinised starch has been reported to be eight times more digestible than ungelatinized starch [7]. The gelatinised starch in the highly porous structure of the bread crumb, coupled with particle breakdown during mastication and the action of salivary amylase, enables easy access for pancreatic amylase to hydrolyse the starch to simple sugars in the human gastro-intestinal tract (GIT) [2,8]. The outcome of this rapid starch hydrolysis in the GIT is a rapid rise in the post-meal blood glucose concentration known as postprandial glycaemia [6]. 

Kumar et al. [9] reported that incorporating nonstarch polysaccharides (NSP) into carbohydrate-rich foods reduced the glycaemic response to them. However, not all NSPs confer similar functionality due to differences in molecular structures and modes of interaction with other components in the food matrix. Generally, when NSPs in food products are mixed with the water phase, they absorb water rapidly and contribute to a rise in digesta viscosity. An increase in the intraluminal viscosity of digesta is postulated to be one of the major factors inhibiting the rate of digestion and the absorption of available carbohydrates [9,10,11]. Several other mechanisms have been proposed, which include: reduced rate of gastric emptying [12], hence the delayed absorption of glucose from the small intestinal lumen [13]; decreased mass transfer by resisting the convective effects of intestinal contractions [14,15,16], and reduced mobility of the fluid layers surrounding the intestinal villi, which increases the resistance of the mucosal diffusion barrier [17,18]. Apart from the effect of viscosity, the formation of a physical ‘barrier’ by certain NSPs (e.g., guar gum), which hinders starch digestion, has also been proposed by Brennan et al. [19]. It is worth noting that different NSPs show different degrees of effectiveness in reducing the glycaemic response. A study by Hardacre et al. [20] showed that the amylolysis of gelatinised starch was not affected by wheat fibre but All-bran^®^ fibre, and, particularly, guar gum showed a significant decrease in the amylolysis of gelatinised starch. The authors suggested the sequences of certain NSPs could inactivate amylase activities. From all these studies, it is evident that the incorporation of NSP in wheat bread formulation remains a plausible strategy to lower bread glycaemic potency. 

It is worth noting that previous research has reported extensively on the effect of hydrocolloids on bread’s functional properties. However, there are no in-depth determinations of glycemic potency in bread containing hydrocolloids. In this study, we investigated the effect of incorporating three types and levels of NSP in a wheat bread formulation. Food grade NSPs, namely xanthan gum, lambda-carrageenan and psyllium husk were evaluated. The effect of NSP and the requirement for additional water for dough development was first determined before the preparation of bread dough formulations. The physical properties of the bread samples in terms of loaf volume and firmness over a two-day storage duration were determined. The digestibility of carbohydrates based on in vitro simulated small intestinal glucose release was used to assess the glycaemic potency of the bread samples. We hypothesized that wheat bread functionality would be altered by the selected NSP due to the potential physico-chemical interactions between NSP and the bread dough structures. We also hypothesised that glucose release during in vitro digestion would be affected to different extents by the types and concentrations of different NSPs incorporated into the breads. Considering the high consumption of bread throughout many parts of the world, this study attempts to provide further knowledge in designing food systems that can play a significant role in the prevention and management of Type 2 diabetes. 

## 2. Materials and Methods

### 2.1. Bread Formulation

The bread formulation consisted of wheat flour, water, yeast, salt and NSP. The wheat flour (Prima Pharaoh flour) consisted of approximately 72% carbohydrate, 12% *w*/*w* protein, 0.40% ash and 14% moisture and it was provided by Prima, Singapore. Instant dry yeast (Saf-Instant, Lesaffre, Marcq-en-Barœul, France) was purchased from NTUC FairPrice, Singapore. The NSPs used were xanthan gum (KELTROL^®^, CP Kelco, Atlanta, GA, USA), lambda-carrageenan (GENUVISCO^®^ CSM-2, CP Kelco) and psyllium husk (Origins Healthfood). 

With the different types and concentrations of NSP introduced in each bread formulation, it was necessary to adjust the amount of water to adequately hydrate the flour for optimal dough development. Farinograph (doughLAB 2500, Perten Instruments, Stockholm, Sweden) analysis, based on the standard AACC International Method 54-21.02 (Rheological Behaviour of Flour by Farinograph: Constant Flour Weight Procedure), was carried out for each formulation consisting of only flour and NSP to determine the amount of water required to obtain a torque of 500 ± 25 FU, which is regarded as optimal for dough development. Each Farinograph analysis required 300 g of flour with the respective amount of NSP, namely 0, 1.0, 3.0 or 5.0% (based on flour weight). Water was added using an automatic water dripping system to the NSP–flour samples, followed by mixing at 30 °C and 63 revolutions per minute (RPM) for 45 min. The amount of water required for each sample to achieve the required dough strength (indicated as torque in Farinograph Units, FU) was recorded. Based on the data obtained from the farinograms (graphs not shown), the formulations of the bread samples (based on 600 g wheat flour each) were calculated, as shown in Table 1.

The bread dough was prepared using the straight dough method (described below), with the aid of a stand mixer (KitchenAid 6.9L Professional Stand Mixer, Benton Harbor, MI, USA) fitted with a spiral dough hook. The required amount of water in each formulation was split into 20% room temperature (~20 °C) and 80% chilled (~12 °C) portions. Instant yeast was hydrated and dispersed in the room temperature water for 5 min before use. All the other dry ingredients were preblended in the mixer bowl at speed 2 for 1 min followed by the addition of the prehydrated yeast and chilled water. Mixing was conducted for 2 min at speed 2 before increasing to level 4 for another 12 min. The dough mixing regime (duration and speed) was consistent for all formulations. The mixing regime was established based on adequate gluten formation by gently stretching a dough sample until a thin elastic dough film could be formed.

The mixed dough was removed from the mixer bowl, formed into a ball, wrapped with a thin polyethylene film and left to rest for 5 min at ~25 °C to ‘relax’ the gluten network. The dough was then split into three portions of 300 g each, shaped and transferred to a proofer oven (Kolb Huizhou Ltd., Huizhou, Guangdong, China) at 30 ± 2 °C and 85 ± 2% humidity (% RH). After ~60 min of proofing, the dough samples were gently kneaded by hand to remove large bubbles formed during the fermentation. Each dough sample was shaped before being transferred to a bread pan for a second round of proofing for ~60 min. This was followed by the baking of the dough samples at 210 ± 2 °C for 35 min in a deck oven (Sveba Dahlen, Fristad, Västra Götaland, Sweden). After baking, the bread samples were allowed to cool for approximately two hours prior to conducting further analysis.

### 2.2. Loaf Specific Volume Measurement

Specific loaf volume was determined using the solid displacement method (AACC International Standards (10-05.01, Guidelines for Measurement of Volume by Rapeseed Displacement). Briefly, a container of known volume was weighed and filled with seeds while the container was constantly tapped. Once the container was filled, the top surface was levelled and the entire container was weighed. The weight of the seeds and the volume of the container were used to calculate the bulk density of the seeds. Loaf volume was calculated from the volume of seeds displaced by the bread loaf. Loaf specific volume (cm^3^/g) was calculated by dividing the loaf volume (cm^3^) by the weight (g) of the loaf. Measurements were conducted in triplicate.

### 2.3. Visual Observation of Crumb Appearance

Bread samples were cut into ~25 mm slices. The appearance of the bread crumb was visually assessed for the size and distribution of the air cells of different bread samples.

### 2.4. Moisture Analysis of Bread Crumb

The moisture content of the bread crumb was measured on Day 0, 1 and 2 after baking. All bread samples were sealed in Ziplock bags and kept in airtight containers at ambient conditions (~25 °C). Moisture analysis was carried out by a halogen moisture analyser (HE53, Mettler Toledo, Columbus, OH, USA) with ~4 g of sample dried at 105 °C under automatic operating conditions. Measurements were conducted in triplicate.

### 2.5. Bread Firmness

Bread firmness was measured on Day 0, 1 and 2 after baking using a texture analyser (TA-XT Plus C-, Stable Micro Systems, Godalming, Surrey, UK). For each bread sample, the crust was removed and the crumb was cut into pieces of 25 mm (height) × 30 mm (length) × 30 mm (width). The bread pieces were sealed in Ziplock bags which were kept in an airtight container and stored at 25 °C (ambient conditions) before texture analysis was conducted. The texture analyser was equipped with a 5 kg load cell and a 75 mm aluminium compression platen (P/75, Stable Micro Systems, Godalming, Surrey, UK). The settings for the measurement were based on 5 g trigger force, pre-test and post-test speeds of 1 mm/s and 2 mm/s, respectively, and 70% strain. Triplicate measurements were conducted for each sample.

### 2.6. In Vitro Method for Determination of Glycaemic Potency of Bread Samples

#### 2.6.1. In Vitro Digestion

The rate of in vitro amylolysis of the bread samples was measured as the rate of the appearance of glucose using an in vitro simulated small intestinal digestion model reported by Akila, Mishra, Hardacre, Matia-Merino, Goh, Warren and Monro [2]. The in-vitro model has been shown to closely mimic in vivo blood glucose response, as it accounts for both glycaemic glucose equivalent (GGE) release from food adjusted to realistic portion size as well as associated systemic blood glucose clearance [21]. The bread loaf was sliced and stored in an airtight container at −18 °C overnight (~18 h). The samples were thawed for approximately two hours at 25 °C (ambient conditions) before carrying out in vitro digestion.

For the in vitro digestions, 5 g of each bread crumb sample (dry weight basis), was added to 60 mL of deionised water and homogenised using a T18 digital Ultra-Turrax^®^ (IKA^®^-Werke GmbH & Co.KG, Staufen, Germany) fitted with an S18N-19G dispersing tool, operating at 6000 RPM for 2 min. The respective homogenised mixture was transferred into a 250 mL Duran^®^ screw cap glass bottle. A control sample was included without any bread sample. Digestion was conducted in two phases—an initial nonamylolytic phase at pH 2.5 that simulated gastric peptic digestion, followed by an amylolytic phase at pH 6.5 that simulated small intestinal digestion.

The gastric phase commenced with the adjustment of the sample mixture to pH 2.5 using 1 M HCl. Next, 2 mL of a solution of 10% (*w*/*v*) pepsin (P7000, Sigma-Aldrich, Burlington, MA, USA; ≥250 U/mL) dissolved in 0.05 M HCl was added. The mixture was stirred at 130 RPM and 37 °C for 30 min (MaxQ 6000, Thermo Fisher Scientific, Waltham, MA, USA). The small intestinal phase was initiated by neutralising the gastric HCl with 4 mL of 1 M sodium bicarbonate (E5420, GCE Laboratory Chemicals, Singapore) and 10 mL of 0.1 M sodium maleate buffer (pH 6). A 0.5 mL sample (time zero) of the suspension was transferred and thoroughly mixed in 2 mL of chilled absolute ethanol.

The amylolysis of the mixture began upon the incorporation of 0.2 mL of amyloglucosidase (E-AMGDF, Megazyme, Bray, Wicklow, Ireland; 3260 U/mL) and 2 mL solution of 5% (*w*/*v*) pancreatin (P7545, Sigma-Aldrich; 8 × USP specifications) dissolved in 0.1 M sodium maleate buffer (pH 6). The mixture was made up to 110 mL volume with 30 mL of deionised water and incubated at 37 °C with continuous stirring (130 RPM). An aliquot of 0.5 mL sample was successively taken at 10, 20, 30, 40, 60, 120 and 180 min from the initiation of digestion, and the amount of glucose released from starch hydrolysis was determined. Each of these samples was thoroughly mixed with 2 mL of chilled 99.9% ethanol (Chemtech Trading, Singapore) to stop the enzymatic activity. All samples (inactivated samples) were then stored at 4 °C before glucose quantification. All analyses were performed in duplicate.

#### 2.6.2. Quantification of Glucose Released

The products of in vitro starch digestion (glucose, maltose, dextrins) that would be absorbed as glucose in vivo were measured after secondary amyloglucosidase digestion by the dinitrosalicylic acid (DNS) colourimetric method for reducing sugars, as reported by Akila, Mishra, Hardacre, Matia-Merino, Goh, Warren and Monro [2]. The DNS assay determines glucose equivalents in solution. From the glucose concentration, the glycaemic index (GI) was calculated. Since nearly all the available carbohydrates digested from the bread samples were glucose (GI = 100%) derived from starch, adjustment for other sugars of lower GI, such as sucrose or fructose, was not required. To quantify the glucose content, a glucose standard curve was first produced using standard glucose solutions at concentrations of 0, 5, 10 and 20 mg/mL

In the DNS method, the samples (enzyme inactivated samples) were first centrifuged at 1000 relative centrifugal force (RCF) and 25 °C for 10 min to remove any particulate matter. Subsequently, 0.05 mL of the sample supernatant or glucose standard was added to 0.25 mL solution of 1% (*v*/*v*) amyloglucosidase (E-AMGDF, Megazyme; 3260 U/mL) and 0.05% (*w*/*v*) invertase (I4504, Sigma-Aldrich; ≥300 units/mg solid) dissolved in 0.1 M sodium acetate buffer (pH 5.2). The mixture was then incubated in a water bath at 37 °C for 15 min to convert all saccharides to glucose. Reducing sugars were then measured by adding 0.75 mL of DNS mixture (containing a 1:1:5 mixture of 0.5 mg/mL glucose solution, 4 M sodium hydroxide and DNS reagent) and heating at 95 °C for 15 min (note that the DNS reagent consisted of 10 g of 3,5-dinitro salicylic acid dissolved in 1.0 L of a solution of 300 g Na-K tartrate and 16 g NaOH). The samples were then cooled in an ice bath, followed by the addition of 4 mL of deionised water. The samples were thoroughly mixed using a vortex mixer. The absorbance of each sample was measured in duplicate at 530 nm with a Cary 60 UV-Vis Spectrophotometer (Agilent Technologies, Santa Clara, CA, USA).

#### 2.6.3. Derivation of Glycaemic Potency of Bread Samples

The amount of glucose released was expressed as GGE per 100 g of bread (based on 72% carbohydrate from the flour in bread and on a dry weight basis). Data analysis was performed using an Excel spreadsheet (Microsoft Corp, Redmond, WA, USA). The predicted relative glycaemic response of the bread samples was estimated by considering the cumulative apparent glucose disposal (GD). The metabolic GD that accompanies the release of sugars during in vivo digestion was estimated for each sampling time using Equation (1):

GD rate = 0.0135x + 0.0232(1)

where x is the quantity of GGE released by 100 g of bread at the 40 min mark of the in vitro digestion [22]. The cumulative apparent GD value was subtracted from the cumulative GGE value at each time point to give the net GGE value (i.e., theoretical excess of blood glucose absorption over glucose disposal that gives the in vivo glycaemic response) at each sampling interval. The net GGE value represents the contribution of the released glucose to the glycaemic response. The net GGE values were plotted against time to obtain a simulated glycaemic response curve. Ten minutes were added to the in vitro digestion time to consider the delay between the consumption of the food and the onset of the in vivo glycaemic response [22]. The incremental area under the blood glucose response curve (IAUC) of the net GGE curve was calculated using a trapezoidal summation technique. The IAUC represents the contribution of starch in the breads to the glycaemic response. The relative glycaemic potency (RGP), in terms of GGE of the test samples, was determined on an equal weight basis (100 g of bread) by comparing the IAUC of each test sample with that of the reference sample of known RGP. It is based on Equation (2):

RGP_test sample_ = IAUC_test sample_/IAUC_reference sample_ × RGP_reference sample_. (2)

The RGP of the reference sample was estimated from its available carbohydrate content and an assumed GI value of 70 for white bread [22,23]. This was determined using Equation (3):

RGP_reference sample_ = % available carbohydrate_reference_
_sample_ × 70. (3)

The available carbohydrate content was taken as the amount of GGE released at the 120 min mark of the in vitro digestion, expressed as a percentage in 100 g of bread. Additionally, rapidly digestible starch (RDS) is the major contributor to glycaemia and may be used to predict the glycaemic response [24]. The RDS content of the bread samples was also compared. It was assessed as the proportion of starch converted to glucose after 20 min of in vitro digestion.

### 2.7. Determination of NSP Leaching

During the consumption of bread, NSP in the bread may diffuse out from the bread matrix during the formation of bolus and chyme when the bread is mixed with saliva and the intestinal fluids, respectively. The extent of NSP leaching depends on the degree of NSP entrapped within the bread matrix. If NSP is leached from the bread matrix, an increase in chyme viscosity can be expected.

Viscosity measurements were used to determine the extent of NSP-leaching from the bread crumb by using a rheometer (MCR 102, Anton Paar GmbH, Graz, Austria) coupled with a cylindrical double-gap measuring system (DG26.7/T200/AL, Anton Paar GmbH, Austria). Each sample (10% *w*/*w*) was prepared by homogenising 4 g of bread (on a dry weight basis) with 36 g deionised water using a T18 digital Ultra-Turrax^®^ (IKA^®^-Werke GmbH & Co.KG) and an S18N-19G dispersing tool at 6000 RPM for 2 min. The mixture was stirred at 250 RPM and 37°C for 30 min and centrifuged at 6000 RCF at 37 °C for 10 min. The viscosity of the supernatant was obtained based on shear rates from 0.1 s^−1^ to 1000 s^−1^ at 37 °C. The viscosity curves generated were compared with pure NSP solutions at 0.46% *w*/*w* (based on the highest level of NSP used in the bread formulations), which is the total concentration of NSP in the bread to represent the complete leaching of the NSP from the bread matrix.

### 2.8. Statistical Analysis

All measurements were carried out in duplicate unless otherwise stated. One-way ANOVA analysis was carried out using Minitab software (version 18) and a comparison of the means was accomplished by Tukey’s test at a 95% confidence interval.

## 3. Results and Discussion

### 3.1. Rheological Behaviour of Dough

**Figure 1:**
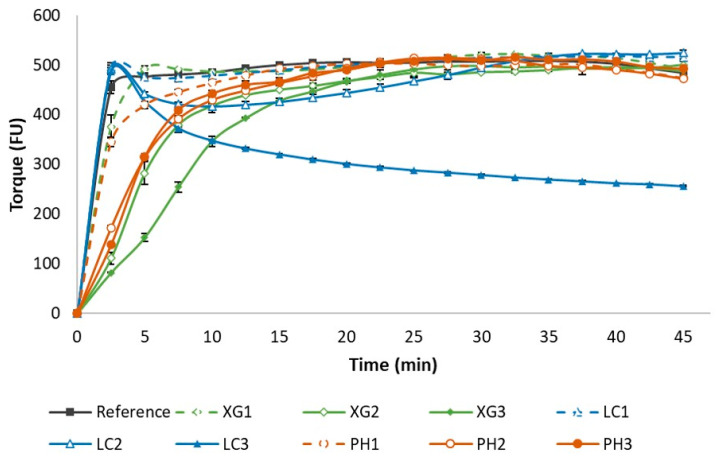
Farinographs show dough development of flour samples, the control and flour with xanthan (XG), lambda-carrageenan (LC) and psyllium husk (PH) addition at three concentrations of 1.0, 3.0 and 5.0% *w*/*w* based on flour weight (denoted as 1, 2 and 3, respectively) over a 45 min mixing regime. Error bar represents mean ± standard deviation, *n* = 3.

Figure 1 shows the farinograph analysis of flour samples containing xanthan gum, lambda carrageenan and psyllium husk, each at 1.0%, 3% and 5% (based on flour weight), respectively. The reference sample was without NSP addition.

From the farinograph analysis, the amount of water needed for each flour–NSP combination based on the target torque of 500 ± 25 FU ranged widely between ~38 to 49% *w*/*w* (see Table 1) or ~65 to 106% *w*/*w* based on flour weight. This meant that the incorporation of NSP required an increased amount of water for adequate dough development. Based on the highest NSP concentration (5% *w*/*w* based on flour weight) for each NSP type reported in this study, the amount of water added increased from ~65% (based on flour weight) for the reference to ~72%, ~87% and ~105% for LC3, XG3 and PH3, respectively. These results indicated that the amount of water needed was not only dependent on NSP concentration but also the water holding capacity of each NSP type.

During the first 10 min of mixing, a drastic increase in the torque was observed (Figure 1) across all samples as hydration of flour and other dry ingredients took place to form a sticky viscoelastic mass. As the wheat gluten becomes hydrated, the formation of the gluten network in the dough started to occur. Optimal dough strength was achieved between 20–35 min of mixing, with the majority of the samples showing stable dough development over the 45 min mixing duration. From Figure 1, the rate of dough development was fairly similar for the reference sample and various NSP samples with the lowest NSP concentration (XG1, LC1 and PH1). However, at higher NSP concentrations (XG2, XG3, PH2, PH3), the rate of dough development appears to be delayed, probably due to the NSP interfering with gluten development. Samples with the highest xanthan gum concentration (XG3) required a longer mixing time to reach optimum dough strength as compared to XG2. In the case of samples containing psyllium husk, no obvious difference was noted between samples PH2 and PH3. Overall, the results showed that a longer mixing time was needed with the incorporation of xanthan and psyllium husk to facilitate sufficient gluten network development as the NSP and wheat gluten competed for water. An unusual trend was noted for lambda-carrageenan (LC3). Firstly, the initial slopes showing the rate of dough development during the first three minutes were like the reference sample regardless of the concentration of lambda-carrageenan. However, the viscosity of LC3 decreased by about 40% after ~10 min of mixing followed by a more gradual decrease as mixing continued. In the case of LC2, the decrease in dough strength was less drastic, but interestingly, with prolonged mixing, dough strength showed complete recovery after 30 min of mixing. A possible explanation for this is that during the first 3 min of mixing, the hydration of ingredients occurred with gluten absorbing sufficient water to initiate a gluten network formation. However, once lambda-carrageenan is sufficiently hydrated, the NSP chains rapidly disrupt the gluten network, resulting in a drastic decrease in dough strength, forming a sticky dough. In the case of LC2, the formation of the gluten network appears to be disrupted by the hydrated lambda-carrageenan molecules during the intermediate stage of mixing, but the prolonged mixing facilitated interactions among gluten chains for optimum gluten network formation to occur. These results indicated that the selection of mixing regime for dough formulations containing NSP is an important consideration as it has been shown to significantly impact dough development, likely due to competition for water by both the gluten proteins and NSP. Overall, the farinograph analyses obtained for different formulations showed the necessity of adjusting the amount of water for formulation with NSP addition for sufficient dough development.

### 3.2. Loaf Specific Volume

**Figure 2:**
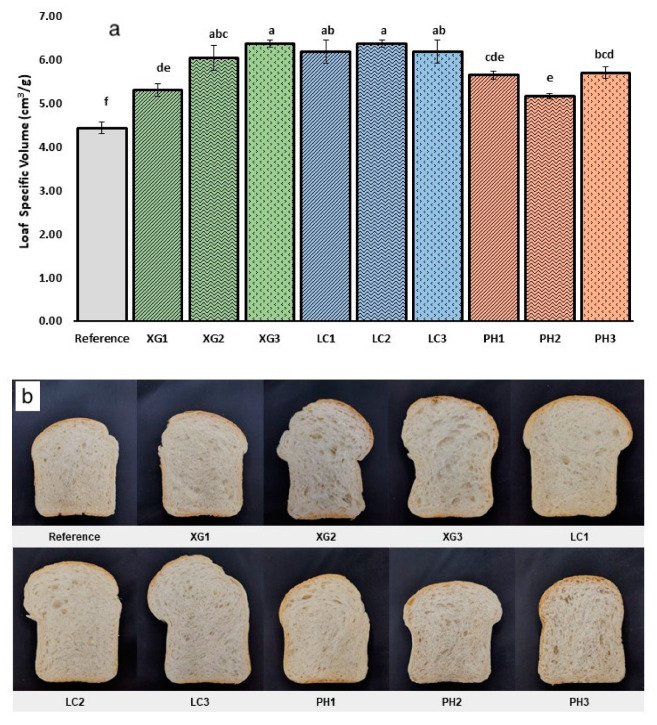
(**a**) Bar graph (**top**). Loaf specific volume across bread formulations. Error bar represents mean ± standard deviation, *n* = 3. Different letters represent significant difference (*p* < 0.05). (**b**) Photographs (**bottom**) indicate the crumb appearance of the bread samples.

The loaf specific volume and crumb appearance of the bread samples are shown in Figure 2.

All samples with added NSP showed significantly increased loaf volume as compared to the reference sample. The increase in loaf volume could be due to the degree of hydration. This occurrence was also reported in gluten-free bread [25]. However, we observed that the loaf volume did not increase further even at higher levels of NSP despite the increase in hydration. Our data concur with Culetu et al. [26], in that there is no correlation between the hydrocolloid concentration and the bread volume. Among the formulations with NSP, the addition of xanthan gum (XG1, XG2 and XG3) resulted in an increase in loaf specific volume by ~20, 36 and 43%, respectively, with increasing NSP concentration. Bread formulations with lambda-carrageenan (LC1, LC2 and LC3) also showed significantly increased loaf volume (*p* < 0.05) across all three concentrations by ~40% across all three concentrations in comparison to the reference sample. An increase in specific loaf volume was previously reported for bread formulation containing lambda carrageenan and other water-soluble NSPs. This was attributed to the increased strength and elasticity of the gluten–starch network with the addition of NSP [27,28]. Similarly, the addition of psyllium husk (PH1, PH2 and PH3) also showed a significant increase in loaf volume by ~27, 17 and 29%, respectively, as compared to the reference sample. However, this result seems to differ from a prior study conducted by Man et al. [29], who reported a decreased loaf specific volume with increasing psyllium husk addition of between 5% to 15% *w*/*w*. The authors attributed the decrease in loaf volume to the dilution of gluten content caused by psyllium fibre. The difference in the results could be due to multiple factors, such as different mixing times and shear, the extent of available water for both NSP and gluten and characteristics of other ingredients present in the formulation. Based on our results, the presence of NSP with additional water compensation in a well-developed gluten dough could strengthen the gluten network, possibly by providing an improved plasticising effect, reducing regions of highly compact gluten–gluten intermolecular interactions [27] and improving dough extensibility. Mironeasa and Codina [30] found that bread specific volume in psyllium enriched wheat bread decreased when the level of psyllium was increased. However, the decrease in specific volume was reduced when the hydration level was increased. Moreover, the presence of water-soluble NSP could potentially contribute to an additional polymeric network formation within the dough. All these factors could lead to a stronger, more extensible viscoelastic structure that facilitates improved gas retention and loaf volume during proofing and baking.

In terms of crumb structure, higher concentrations of xanthan gum, lambda-carrageenan and psyllium husk appeared to contribute to the decreased uniformity of the crumb structures with a tendency towards larger air cells (Figure 2b). Such variations in crumb structures could contribute to the different mechanical and organoleptic properties of the bread [31].

### 3.3. Moisture Content of Bread Crumb

**Figure 3:**
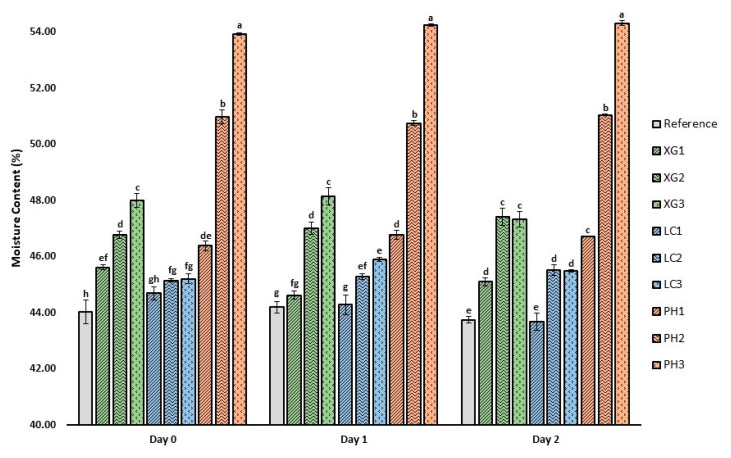
Moisture content of bread samples over three days of storage at room temperature (~25 °C). Error bars represent mean ± standard deviation, *n* = 2. Different letters represent significant difference (*p* < 0.05) within the same day (i.e., Day 0, 1 or 2).

The moisture content of the bread samples over three days (Day 0, 1 and 2) is shown in Figure 3.

With an increased amount of water incorporated into the bread formulations containing NSP, a corresponding increase in the moisture content of baked bread can be expected due to the water-binding capacity of NSPs [32]. In general, there is a significant increase in moisture content with the incorporation of NSPs. The increase in moisture content is NSP concentration-dependent, where an increase in NSP concentration within the bread formulation led to a proportional increase in moisture content. However, the extent of the increase varies with the type of NSP and its ability to absorb water, with lambda-carrageenan (1.5%, 2.5%, 2.7%) showing the mildest change, xanthan gum showing moderately high water absorption (3.6%, 6.2%, 9.0%) and psyllium husk (5.3%, 15.7%, 22.5%) being the most pronounced. The high water-holding capacity of psyllium husk is attributed to the high proportion of hemicellulose, which is composed of a xylan backbone linked with arabinose, rhamnose and galacturonic acid units [33,34]. Leon, Ribotta, Ausar, Fernandez, Landa and Beltramo [27] reported that psyllium husk contributed to higher retention of strongly bound water in gluten-free bread compared to xanthan, guar gum and hydroxypropylmethylcellulose. Our study also showed that during storage, there were minimal changes in the moisture content of bread samples with each respective NSP over the two-day storage duration. This could be explained by the good water-binding capacity of these NSPs, which slowed down moisture loss during the storage [35].

### 3.4. Firmness of Bread

**Figure 4:**
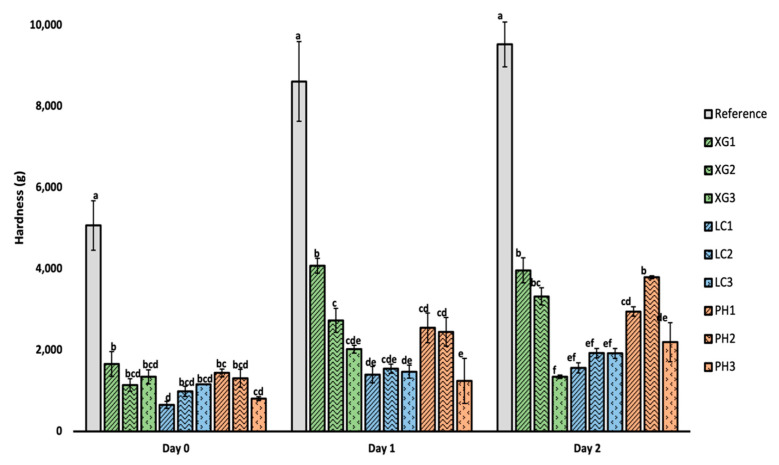
Hardness of bread at day 0, 1 and 2 storage duration at room temperature (~25 °C) obtained by compression test. Error bar represents mean ± standard deviation, *n* = 3. Different letters represent a significant difference (*p* < 0.05) within each storage duration (i.e., Day 0, 1, 2).

The firmness data of bread samples on day 0, 1 and 2 are shown in Figure 4.

Clearly, all bread samples with the addition of NSP showed significantly lower hardness as compared to the reference sample. With storage, bread hardness generally increased over time of storage for the reference sample largely due to starch retrogradation [36]. A similar trend was observed for the bread samples containing each NSP type. However, in contrast to the reference, bread samples with NSP were still less than half the hardness of the reference bread after the second day of storage. We had initially attributed the lower hardness values to the higher moisture content in bread and higher specific loaf volume in bread containing NSP. However, both moisture and specific loaf volume showed relatively weak correlation coefficients with R-sq values of ~0.13 and ~0.63, respectively, suggesting a relatively mild impact of these factors on lowering the hardness of the NSP-incorporated bread. Another hypothesis is that the addition of NSP potentially retards staling by delaying starch retrogradation [35]. While retrogradation in bread is complex, it has been broadly attributed to the recrystallization of amylopectin. Such recrystallization has been widely reported as the main cause for the increased hardness of bread over time. Mandala [37] explained the slowdown in starch retrogradation could be due to the interactions between the hydrocolloids and the amylopectin. The interactions could inhibit the formation of crystalline structures or the stabilisation of water molecules by the hydrocolloid polymers, which deprives the amylose or amylopectin of water for crystallisation.

Concerning NSP concentration, increasing the level of NSP did not have any significant effect on hardness at Day 0. However, the effect of NSP concentration appeared to be more pronounced after Day 1 and 2 of storage duration. Such effect was particularly pronounced in samples containing xanthan gum or psyllium husk, with the highest concentration showing the lowest hardness upon storage. A study carried out by Abdullah et al. [38] also reported that bread enriched with 5% psyllium husk produced a significantly softer bread texture. For bread with lambda-carrageenan, no significant difference in hardness was observed at different concentrations for each of the storage intervals.

### 3.5. Glycaemic Potency of Bread Samples

**Figure 5:**
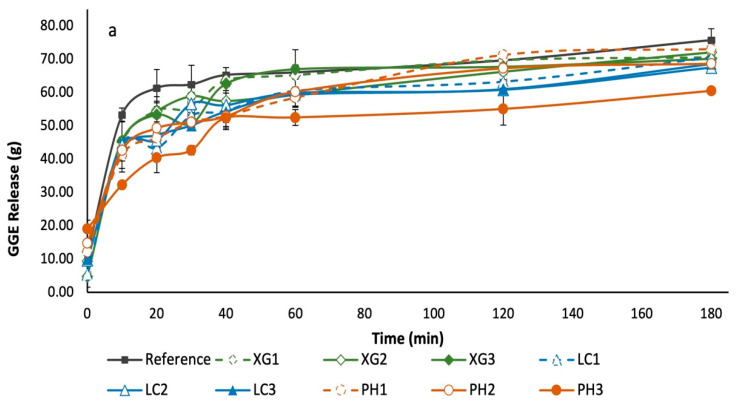

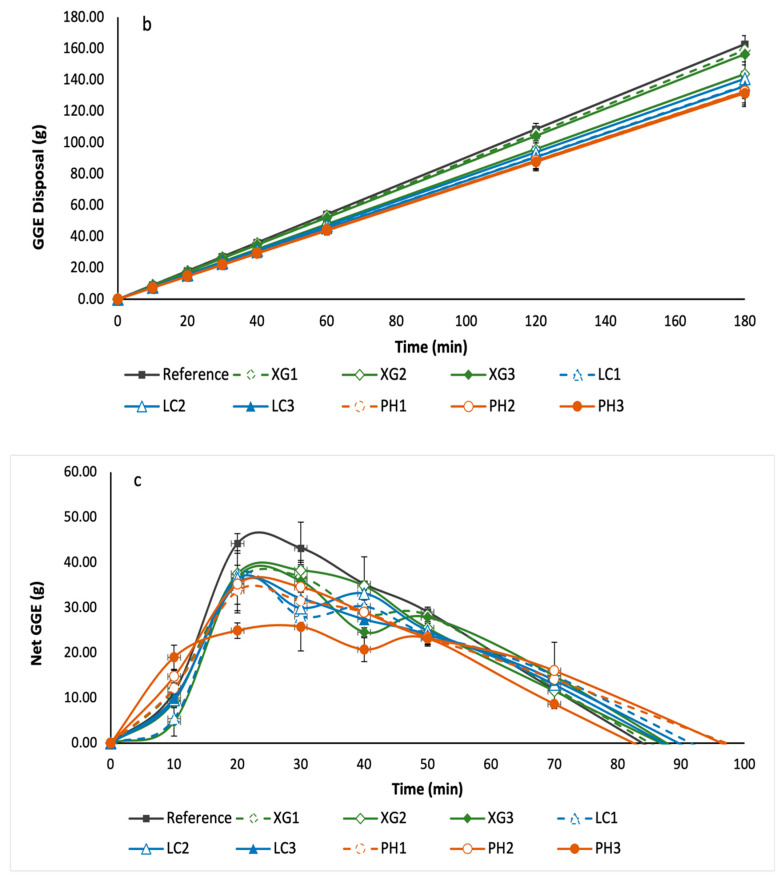
(**a**) GGE release curves show the overall accumulated glucose release over time; (**b**) GGE disposal curves show theoretical accumulated glucose disposal over time; and (**c**) net GGE curves obtained by subtracting GGE disposal from GGE release. All data are based on a dry weight basis of bread samples.

The in vitro digestibility profiles of the bread samples, from which the GGE release and expected GGE disposal were calculated, yielding net GGE curves by difference, are shown in Figure 5. From the profiles, the IAUC, RGP and RDS values of the various bread samples were derived (Table 2). The IAUCs were determined as the areas under the net GGE curves calculated from the digestion profiles of the different bread samples. From the IAUC data, the RGP values of the respective samples were calculated. The RGP value indicates the amount of glucose equivalent in 100 g of bread in terms of the predicted glycaemic response. As for the RDS content of the bread samples, it was derived as the proportion of starch converted to glucose following 20 min of in vitro digestion.

Glucose release during in vitro digestion was quantified through the use of the DNS colorimetric method, where values were derived with the use of a glucose standard curve (y = 0.1007x + 0.1848, where y is the absorbance at 530 nm and x is mg glucose/mL digestion mixture). Subsequently, these values were converted to g GGE/100 g dry weight bread sample (taking into account the respective dilution factors). These values were plotted against the in vitro digestion time to provide GGE release curves (Figure 5a). An apparent glucose disposal (GD) rate was derived using the formula GD rate = 0.0135GGE_40min in vitro_ + 0.02232, which was used to derive GGE disposal for each time point, which was plotted against actual in vitro time, as illustrated in Figure 5b. Subsequently, net GGE values were obtained by subtracting GGE disposal from GGE release to account for blood glucose clearance. Ten minutes were added to the actual in vitro time to allow for the typical in vivo delay in the onset of glycaemic response. The net GGE values are plotted in Figure 5c.

Commonly, GI values are categorised as low (0–55), medium (56–69) and high (>70). The estimated GI values of the bread samples and respective GI categories are listed in Table 3.

With reference to Table 3, the addition of the three NSP was shown to reduce the glycaemic potency of white wheat bread but to a different extent. Increasing the concentration of xanthan gum and lambda-carrageenan did not show any further decrease in the glycaemic potency. However, bread with the highest concentration of psyllium husk (PH3) showed the lowest GI value, within the Low GI category. Overall, different NSP types and concentrations appeared to affect the glycaemic potency of white wheat bread differently. Due to the different physico-chemical properties of each NSP, their interactions with different components (such as starch and gluten) in bread could ultimately influence the rate and extent of hydrolysis of starch within the bread matrix.

### 3.6. Leaching of NSP from Bread

**Figure 6:**
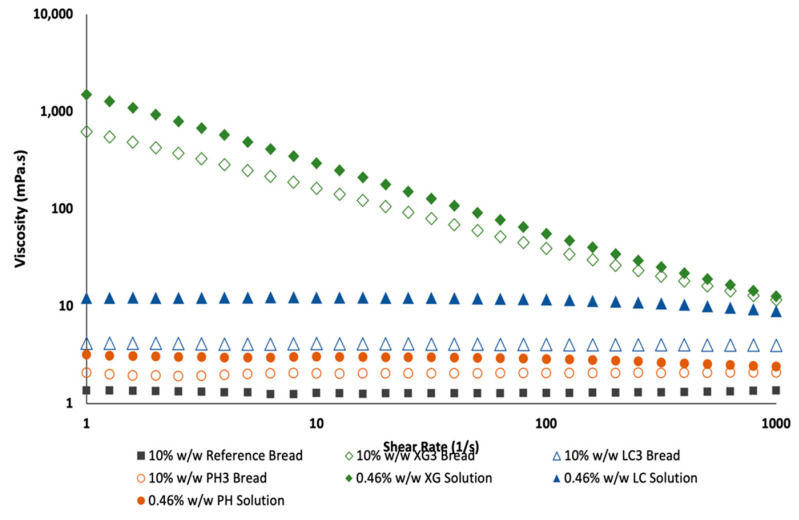
Viscosity curves of supernatant of homogenised bread suspension (10% *w*/*w*) formulated with 5% *w*/*w* of soluble NSP and 0.46% *w*/*w* pure NSP solutions. XG, LC and PH denote xanthan gum, lambda-carrageenan and psyllium husk, respectively. Values are averages of duplicate measurements.

The extent of NSP-leaching from the bread during mastication was assessed by comparing the supernatant of homogenized bread samples (XG3, LC3 and PH3) and NSP solutions prepared based on an equivalent concentration of NSP present in the bread (Figure 6).

The viscosity values of the supernatant solutions obtained from bread samples were lower than the pure respective NSP solutions but higher than the reference sample. This means that some leaching of NSPs from the homogenized bread had occurred. Among the bread samples, the viscosity at shear rates between 10 and 100 s^−1^, typical of shear rates in the gastrointestinal tract [39,40], showed that the bread containing xanthan gum can potentially form a higher chyme viscosity at least 10 times greater (based XG3 with ~60 mPa.s at 50 s^−1^) than bread containing lambda-carrageenan (~4 mPa.s at 50 s^−1^) or psyllium husk (~2 mPa.s at 50 s^−1^). The higher chyme viscosity suggests that the incorporation of xanthan gum in bread formulation can potentially reduce the glycaemic potency. The effect of viscosity in reducing glycaemic potency in white bread has been reported in various studies [15,20,41].

Compared to the reference bread, the addition of psyllium husk (PH3) or lambda-carrageenan (XG3) could be expected to have a low to negligible effect on the ‘chyme’ viscosity and both had viscosity values close to water. It is worth noting that psyllium husk consists of both soluble and insoluble polysaccharide fractions and most of the psyllium would remain entrapped within the solid fraction of the bread matrix. This could imply that the marked decrease in glycaemic potency with PH3 addition (Table 3) may not be attributed to the viscosity of leached polysaccharides. Instead, psyllium husk possibly interacted with the bread matrix that was formed from gluten and gelatinised starch. In addition, as reported previously for xanthan, psyllium husk could restrict starch gelatinization by coating the starch granules or/and affecting amylose interactions in the final network [42]. The distribution of psyllium husk at sufficient concentrations within the bread matrix could then hinder enzymes from rapidly hydrolysing starch distributed in the bread structure during the in vitro digestion process, yielding bread with low GI values. The effectiveness of psyllium in lowering GI has also been reported in gluten-free bread formulation [26]. Other possible effects, such as the direct inhibition of enzyme activity reported in the case of guar gum [43], cannot be ruled out at this stage and would require further validation in future studies. All these possible mechanisms involving NSP can potentially aid in slowing postprandial blood glucose response [39].

## 4. Conclusions

This study investigated the effects of xanthan gum, lambda-carrageenan and psyllium husk utilised in a white bread formulation. The results showed that the incorporation of xanthan gum, lambda- carrageenan and psyllium husk showed some reduction in the glycaemic potency of bread with PH3 (psyllium husk at 5% *w*/*w* on flour weight basis), showing the most pronounced reduction (up to 24%) in GI. The mechanisms by which the reduction in glycaemic potency appeared to be different among different types of NSP. In the case of xanthan gum, the higher viscosity effect of xanthan gum seemed to be the reason for slowing down starch hydrolysis. In the case of psyllium husk, the possible formation of NSP-starch complexes could explain why it was effective in retarding starch hydrolysis. Based on both physical functionality and the overall effect on glycaemia, PH3 yielded the most desirable outcomes with increased loaf specific volume by about 12.6%, reduced bread hardness (by about 67%), and after storage, the PH2 bread was about 50% the hardness of the reference sample, although both the reference and PH2 breads had similar proportional increases in hardness during storage. The selection of a suitable NSP and the understanding of NSP–starch interactions are key considerations for food product developers when formulating foods to achieve the desired organoleptic properties with improved health benefits.

## Figures and Tables

**Table 1:** Formulation of bread doughs containing different concentrations of xanthan gum (XG1, XG2 and XG3), lambda-carrageenan (LC1, LC2 and LC3) and psyllium husk (PH1, PH2 and PH3), respectively. The reference sample is control without added NSP.

Formulation	Ingredients	Flour	Water	Yeast	Salt	NSP	Total
Reference	g	600	387.4	7.2	12	0	1006.6
	%*w*/*w*	59.6	38.5	0.7	1.2	0.0	100.0
XG1	g	600	415.2	7.2	12	6	1040.4
	%*w*/*w*	57.7	39.9	0.7	1.2	0.6	100.0
XG2	g	600	480.6	7.2	12	18	1117.8
	%*w*/*w*	53.7	43.0	0.6	1.1	1.6	100.0
XG3	g	600	525.3	7.2	12	30	1174.5
	%*w*/*w*	51.1	44.7	0.6	1.0	2.6	100.0
LC1	g	600	390.3	7.2	12	6	1015.5
	%*w*/*w*	59.1	38.4	0.7	1.2	0.6	100.0
LC2	g	600	414	7.2	12	18	1051.2
	%*w*/*w*	57.1	39.4	0.7	1.1	1.7	100.0
LC3	g	600	435.3	7.2	12	30	1084.5
	%*w*/*w*	55.3	40.1	0.7	1.1	2.8	100.0
PH1	g	600	426	7.2	12	6	1051.2
	%*w*/*w*	57.1	40.5	0.7	1.1	0.6	100.0
PH2	g	600	528.9	7.2	12	18	1166.1
	%*w*/*w*	51.5	45.4	0.6	1.0	1.5	100.0
PH3	g	600	634.2	7.2	12	30	1283.4
	%*w*/*w*	46.8	49.4	0.6	0.9	2.3	100.0

**Table 2:** Bread formulations and the respective IAUC, RGP and RDS values based on dry weight.

Formulation	IAUC (g.min)	RGP (GGE/100g dry wt)	RDS (%)
**Reference**	1978.42 ± 186.79 ^a^	48.75 ± 1.20 ^a^	61.32 ± 5.57 ^a^
XG1	1781.81 ± 225.49 ^ab^	40.45 ± 5.12 ^ab^	54.58 ± 4.22 ^ab^
XG2	1796.87 ± 24.45 ^ab^	40.79 ± 0.55 ^ab^	54.31 ± 3.07 ^ab^
XG3	1716.59 ± 167.89 ^ab^	38.97 ± 3.81 ^ab^	53.30 ± 3.67 ^ab^
LC1	1713.33 ± 264.11 ^ab^	38.89 ± 6.00 ^ab^	43.50 ± 0.00 ^b^
LC2	1710.49 ± 12.00 ^ab^	38.83 ± 0.27 ^ab^	45.63 ± 1.48 ^b^
LC3	1686.67 ± 134.73 ^ab^	38.29 ± 3.06 ^ab^	47.22 ± 6.51 ^ab^
PH1	1756.26 ± 24.56 ^ab^	39.87 ± 0.56 ^ab^	46.31 ± 2.99 ^ab^
PH2	1855.75 ± 43.61 ^ab^	42.12 ± 0.99 ^ab^	49.31 ± 3.01 ^ab^
PH3	1394.10 ± 50.28 ^b^	31.65 ± 1.14 ^b^	40.40 ± 4.49 ^b^

XG, LC and PH denote xanthan gum, lambda-carrageenan and psyllium husk, where concentrations are shown in increasing order (represented by 1, 2 and 3, respectively). Incremental area under the curve (IAUC), relative glycaemic potency (RGP) and rapidly digested starch (RDS) values are expressed as mean ± SD (*n* = 2); different letters in a column represent a significant difference (*p* < 0.05).

**Table 3:** Estimated GI values and categories of bread formulations based on a dry weight basis.

Formulation	Estimated GI			GI Category
	IAUC	RGP	RDS	
**Reference**	70	70	70	High
XG1	63	58	62	Medium
XG2	64	59	62	Medium
XG3	61	56	61	Medium
LC1	61	56	50	Medium/Low
LC2	61	56	52	Medium/Low
LC3	60	55	54	Medium/Low
PH1	62	57	53	Medium/Low
PH2	66	60	56	Medium
PH3	49	45	46	Low

## Data Availability

Data is contained within the article.
